# Input Complexity Affects Long-Term Retention of Statistically Learned Regularities in an Artificial Language Learning Task

**DOI:** 10.3389/fnhum.2019.00358

**Published:** 2019-10-15

**Authors:** Ethan Jost, Katherine Brill-Schuetz, Kara Morgan-Short, Morten H. Christiansen

**Affiliations:** ^1^Department of Psychology, Cornell University, Ithaca, NY, United States; ^2^Department of Psychology, University of Illinois at Chicago, Chicago, IL, United States

**Keywords:** statistical learning, artificial language learning, second language learning, retention, memory

## Abstract

Statistical learning (SL) involving sensitivity to distributional regularities in the environment has been suggested to be an important factor in many aspects of cognition, including language. However, the degree to which statistically-learned information is retained over time is not well understood. To establish whether or not learners are able to preserve such regularities over time, we examined performance on an artificial second language learning task both immediately after training and also at a follow-up session 2 weeks later. Participants were exposed to an artificial language (Brocanto2), half of them receiving simplified training items in which only 20% of sequences contained complex structures, whereas the other half were exposed to a training set in which 80% of the items were composed of complex sequences. Overall, participants showed signs of learning at the first session and retention at the second, but the degree of learning was affected by the nature of the training they received. Participants exposed to the simplified input outperformed those in the more complex training condition. A GLMM was used to model the relationship between stimulus properties and participants’ endorsement strategies across both sessions. The results indicate that participants in the complex training condition relied more on an item’s chunk strength than those in the simple training condition. Taken together, this set of findings shows that statistically learned regularities are retained over the course of 2 weeks. The results also demonstrate that training on input featuring simple items leads to improved learning and retention of grammatical regularities.

## Introduction

Statistical learning (SL) has been identified as a domain-general cognitive ability that is integral to language processing, acquisition, and evolution (see Armstrong et al., 2017, for an overview). For the purposes of this study, we can define SL as the process by which learners uncover the structure of the input from its distributional properties (Frost et al., 2015). However, little is known about the extent to which statistically learned information is retained over time (see Gomez, 2017, for a review), particularly in adult learners.

Initial studies of SL focused on the rapidity with which human infants could learn from predictable, structured sequences of input (e.g., Saffran et al., 1996). As a result, the literature has remained quite focused on measuring the ability of participants to learn these regularities within a single session, usually with a test-phase following some sort of training. However, there have been only a handful of studies examining the ability of adult participants to retain statistically learned information over long periods of time.

Previous work that has focused on adult SL includes Kim et al. (2009). This study demonstrated that participants implicitly learned the statistical relationships governing a sequence of rapidly presented visual stimuli, and that this learning was retained over the course of 24 h. Other work has shown that adults possess the ability to maintain information about the underlying relationship between visual stimuli in an SL task, with testing periods at 30 min, 1, 2, 4, and 24 h delays (Arciuli and Simpson, 2012). Participants showed no difference in their ability to correctly identify grammatical sequences, suggesting that at least over the course of a day, the information gleaned within a(n) SL task is relatively robustly retained. The authors of this study notably suggest that their findings do not indicate any sort of enhancement in retention for participants who slept between training and test.

Additional research, however, has attempted to examine the role of sleep in the consolidation of associations learned within a(n) SL paradigm, and a few of these findings have a bearing on retention and SL more generally. Although the present study does not seek to examine the effects of sleep on SL, this is perhaps the most well-studied aspect of long-term retention within the literature. Napping appears to improve consolidation, as participants who slept during a 4 h delay period between training and test outperformed those who did not during an auditory discrimination task (Durrant et al., 2011). Interestingly, this enhancement was positively correlated with the amount of slow-wave sleep obtained by the participant. Similarly, researchers have shown that participants who slept were more likely to apply statistically learned constraints in a speech production task (Gaskell et al., 2014). This study also demonstrated a positive relationship between slow-wave sleep and learning effects. In general, it seems that over a relatively brief period of time, knowledge gained in a(n) SL task can be retained, and that this retention may even be enhanced by sleep in some instances. More recently, another sleep study by Frost and Monaghan (2017) demonstrated that participants who underwent a period of sleep between training and test within a non-adjacency SL paradigm outperformed those who stayed awake at both word learning and generalizing the rules of the grammar to new sequences that had not been seen during training.

Two studies in particular stand out as examples of investigations into a more traditional definition of long-term retention and consolidation of sequence learning abilities. Romano et al. (2010) demonstrated that participants seemed to retain sequence-specific learning and general skill effects a year after training on a serial reaction time task (pressing a key corresponding to a target circle’s location as targets appeared on the computer screen). Retention was observed across a variety of training groups: younger adults, older adults, experienced musicians, and video game players. In effect, participants recalled frequent triplets more quickly than they did low-probability control trials, showing a learning effect over the course of the tasks at session one that persisted at session two, 1 year later.

Kobor et al. (2017) attempted to extend these findings in a task designed to test consolidation along with retention, as they were interested in uncovering the core mechanism(s) that underlie long-term memory formation in such a task. This study investigated the role of retroactive interference in forgetting by training participants on a new set of items with an alternate statistical structure 1 day after the initial test session. The second test session in this study, which tested long-term retention of the initially trained patterns, took place a full year later, similar to the Romano et al. (2010) study. Again, the researchers found learning effects for highly frequent items relative to infrequent items, and also found no effect for the potentially interfering materials. An additional test demonstrated that the knowledge gained in this task seemed to be implicit in nature. They took this to mean that long-term memory for statistically learned sequences does undergo a process of consolidation that appears to be robust and resistant to some kinds of interference. Moreover, learning scores were reported as relatively stable between the first session, the interference training session the next day, and the final session a year later.

While the previous two studies demonstrate the persistence of sequence learning abilities over a long stretch of time, they are still limited in a few important ways. First, the tasks in both studies required only visuospatial to motor mappings without any auditory or verbal component, limiting the degree to which their findings might generalize to language itself. Second, and relatedly, the learned sequences did not contain any kind of meaning. While this is a common practice within the SL literature, it limits the study’s ecological validity when it comes to addressing the mechanisms thought to underlie language learning (Morgan-Short, in press). Third, the statistical structure underlying the training items was not very complex in either study, again somewhat undermining the claim that the kinds of relationships learned between items in a sequence are characteristic of those in natural language. Finally, neither of these studies demonstrated a quintessential feature of learning in SL and artificial grammar learning (AGL), the generalization of learned regularities to new items. The test sets in each task contained exclusively items on which participants had already been trained.

A second set of studies that has focused more directly on natural and artificial second language learning has also provided evidence of retention over periods of time. Indeed, the delayed posttest as a measure of retention is not uncommon in second language acquisition research as shown in a meta-analysis (Norris and Ortega, 2000), as nearly half of all studies featured some kind of follow-up test phase more than a week after training. This meta-analysis revealed a robust learning effect (Cohen’s *d* = 1.02) at delayed testing. Specifically addressing the question of second language retention, Morgan-Short et al. (2012a) reported behavioral and neurophysiological evidence of retention over the course of 3–6 months for the same artificially learned language used in this study, Brocanto2. Using an artificial, as opposed to natural, language allowed for control of prior experience and extra-experimental exposure to the second language. Participants in Morgan-Short et al. (2012b) achieved high levels of proficiency (∼95%) by completing three training and practice sessions, in which they completed a total of 36 comprehension and production practice modules comprised of 20 practice items each. When tested for retention 3–6 months later, there was no evidence of a decline in their performance (Morgan-Short et al., 2012a). Because of the extensive practice provided in Morgan-Short et al. (2012b), it is not possible to determine if exposure itself led to retention. Thus, the current study aimed to examine retention of grammatical regularities after shorter periods of exposure to input but without practice. It also leveraged the artificial language paradigm Brocanto2 to control exposure to the second language and to manipulate the input (see below).

Given the frequently described links between SL and language, it would seem likely that the associations learned in commonly used SL paradigms should persist over longer periods of time than we currently have robust evidence for, an issue the current study seeks to address with the hypothesis that participants will retain learned information over the course of 2 weeks. In other words, if statistically learned information truly undergirds our language learning abilities, it must be retained beyond an immediate post-test in the lab.

### Input Complexity Affects the Learning of Statistical Regularities

The idea that learners process the co-occurrence statistics of the input in the service of acquiring more abstract grammatical regularities is not new (Elman, 1990; Altmann, 2002). This processing ability has been proposed to develop as we learn the most basic information available from the input first, as suggested by the “less is more,” hypothesis: that is, beginning to learn without fully developed cognitive abilities could convey an advantage to children (Newport, 1990). This notion has been applied to the process of language learning and has been pointed out as a potential reason for the existence of sensitive periods in language acquisition (Johnson and Newport, 1989; Newport, 1990, 2016). The corresponding idea that “starting small” may be advantageous for learners shares similar longevity within the literature (Elman, 1993; Elman et al., 1996), and emphasizes the possible benefit that reduced complexity within the learner’s input (e.g., in terms of length or syntactic complexity) has on learning.

Although the evidence for these hypotheses has subsequently become somewhat less straightforward (for example, see Rohde and Plaut, 1999; Siegelman and Arnon, 2015), new research is emerging that, within the context of artificial language learning, participant performance may benefit from training that becomes progressively more challenging (Kersten and Earles, 2001; Lai and Poletiek, 2011). A recent study has shown that starting small leads to better learning of recursive structures, with the primary facilitation coming from a gradual increase in stimuli complexity rather than simply the effect of reduced length (Poletiek et al., 2018).

Other work has also shown that artificially biasing the kinds of chunks that adults form to be more simplified can lead to improved learning in a Hebb-repetition paradigm (Smalle et al., 2016). Smalle et al. (2018) expanded upon this idea by showing that children exhibited better retention of implicitly learned phonological sequences within a Hebb-repetition task than adults in a longitudinal design with a year between the first and last test sessions. This study demonstrated the long-term retention of input containing probabilistic dependencies, highlighting the importance of chunking as a potential factor for both learning and retention within such paradigms. However, this study left open the relative importance of input complexity on learning, and did not test for generalization of learned knowledge to novel test items.

Taken in conjunction with other recent ideas, chunking can be seen as an integral component of the SL process as it applies to language (Isbilen and Christiansen, 2018; Christiansen, 2019). Rapidly recoding and compressing information by chunking may allow learners to more efficiently process input, and to do so at higher levels of abstraction. In fact, stronger learners may show a decreased reliance on surface-level fragment information when tested due to the fact that they have already used that information to internalize the higher-order regularities, and no longer rely on them as a crutch.

### The Current Study

The present study seeks to examine the different ways in which learners retain knowledge about the grammatical regularities of an artificial language, Brocanto2 (Morgan-Short et al., 2010, 2012b), through the process of SL. To that end, we conducted original analyses of unpublished data from Brill-Schuetz (2016). In this study, training conditions differed by the amount of exposure to complex stimuli presented in the training, where complexity was related to the cognitive demands needed to process an item. In the Simple condition, half of the participants in this study received a more simplified set of training items generated by the grammar. This manipulation attempted to mimic the constraints placed on young learners by the simplified input they tend to receive (Cameron-Faulkner et al., 2003). Training sets with progressively increasing difficulty have been used in past AGL and SL studies for similar reasons (e.g., Conway et al., 2003; Christiansen et al., 2012; Poletiek et al., 2018). Those in the simple training condition were eventually exposed to complex items, but the extensive experience they received with simple items before moving on to the more complex ones is expected to boost performance in the test phase of the experiment. Therefore, our first prediction is that learners, particularly those who receive simple training, will retain knowledge from training over the span of 2 weeks.

In the Complex condition, the other half of participants received far less training with simplified items prior to exposure to the set of complex items yet obtained the same amount of total experience in terms of number of trials. These participants are thus predicted to have more trouble learning, and subsequently retaining, the rules of the artificial language as they would have insufficient experience processing simple constructions before encountering the more difficult complex items. This may lead them to adopt poor learning strategies, disrupting their extraction of the relevant statistical structure embedded within the sequence. In short, more exposure to simple items (i.e., the simple training condition) should confer an advantage to the learning of the grammar rules that govern Brocanto2 both in the short- and long-term (Brill-Schuetz, 2016).

We are also interested in finding out how the different training groups approach the task of endorsing items as grammatical, by looking into what features of the test items are most relevant to such judgments. While the Brocanto2 artificial language learning paradigm was not designed to test SL, the underlying distributional information embedded within its dependencies offers a potential window into the ways that learners use statistical regularities to learn language. Examining endorsement strategies is expected to provide insight into what each group of participants retained from the task across both sessions, and what kinds of information they are sensitive to. The specific cues that participants rely on to make grammaticality judgments might vary between the training groups, and if participants in the complex training condition show the reduced sensitivity to the grammatical regularities of the language that we predict, we hypothesize that they will instead be found to rely more on fragment information, such as chunk strength. On the other hand, the simple training group will likely not be as distracted by surface-level similarities between training and test items and will rather demonstrate knowledge of the higher-order grammatical regularities.

## Materials and Methods

### Participants

Participants (*N* = 47; Male = 10) were young adult students at a large, Midwestern university, ranging in age from 18 to 24 (*M* = 19.43, *SD* = 1.98). Recruitment for the first session was conducted through a psychology department subject pool where participation earned class credit. For the second session, some participants received additional credit through a subject pool and others received monetary compensation ($5). Selection criteria limited participants to those who had no hearing, learning, or speaking impairments, and to native speakers of English. All participants provided written consent before beginning the study^1^.

The second session took place approximately 2 weeks after the original training session. Although every effort was made to schedule the delayed post-test exactly 2 weeks from the original session, the actual range was between 12 and 14 days from the training session. At this second session, some (*N* = 33) participants also completed an additional battery of cognitive tests.

### Materials

#### Artificial Language

**FIGURE 1:**
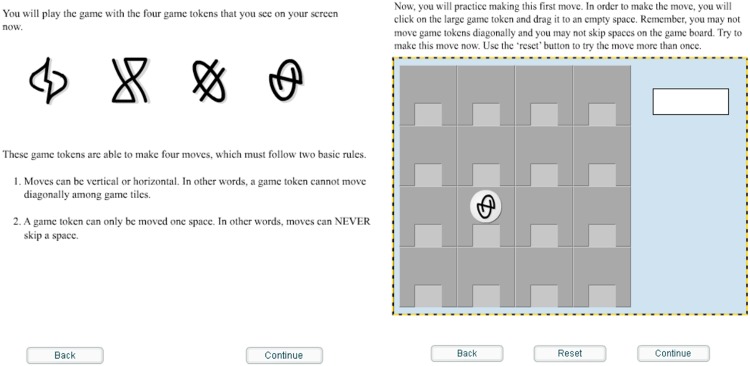
Screenshot of training on the Brocanto2 paradigm. Note that the icons are presented without any outline of a shape around it until it is used as a token on the board game.

The artificial language learned by participants was Brocanto2^2^ (Morgan-Short et al., 2010, 2012b), which was adapted from the original version, Brocanto (Friederici et al., 2002). Brocanto2 follows basic patterns typical of many natural languages and is fully productive; it consists of 14 novel words: four nouns, two adjectives, two articles, four verbs, and two adverbs (see Table 1 for a list of all words and their meanings). The grammatical structure of this language follows a syntactic pattern different from that of English; while English follows a subject-verb-object order, Brocanto2 follows a subject-object-verb order, which is found in languages such as Hindi and Japanese. For example, the Brocanto2 sentence “*Blom neimo lu neep troise li praz zayma”* corresponds to “Blom-piece square the neep-piece round the switch horizontally” and would be translated into English as “The square blom-piece switches with the round neep-piece horizontally.” Participants learned this artificial language in order to play a computer-based game in which the tokens can move according to dictation in Brocanto2 (see Figure 1).

**TABLE 1:** Complete list of words used within the artificial language learning task.

**Word category**	**Brocanto2 word**	**Symbol/meaning**
Noun	Pleck_m_	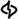
	Neep_m_	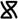
	Blom_*f*_	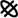
	Vode_*f*_	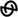
Adjectives	Trois(e_m_/o_*f*_)	Circle
	Neim(e_m_/o_*f*_)	Square
Determiners	li_m_/lu_*f*_	The
Verbs	klin_intran_	Move
	praz_tran_	Switch
	nim_tran/intran_	Capture
	yab_tran/intran_	Release
Adverbs	Noyka	Vertically
	Zayma	Horizontally

**Subscripts denote the gender of each noun and determiner along the corresponding marking for each adjective, and also the transitive nature of each verb. The adjectives described the shape of the area bordering the game piece, such as the circle that can be seen in Figure 1. Table adapted from Morgan-Short (2007).**

**FIGURE 2:**
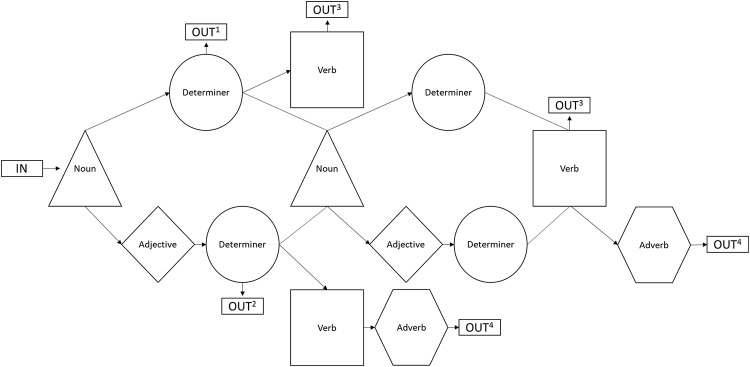
Chart depicting the possible word class combinations of items generated by the Brocanto2 grammar. The superscript at each output classifies the category of phrase or sentence that such a sequence produces; ^1^ denotes a simple phrase (noun + determiner), ^2^ denotes a complex phrase (noun + adjective + determiner), ^3^ denotes a simple sentence (noun + determiner + verb; noun + determiner + noun + determiner + verb), and ^4^ denotes a complex sentence (noun + adjective + determiner + verb + adverb; noun + adjective + determiner + noun + adjective + determiner + verb + adverb.

These sentences could be either simple or complex in nature; simple stimuli were limited to words from three of the word categories (noun, article, verb) and could consist of three to five lexical items. Complex stimuli consisted of words from all five of the categories allowed in Brocanto2 (noun, adjective, article, verb, adverb) and a complex sentence could contain five to eight lexical items (Brill-Schuetz, 2016). For example, the sample sentence given above would be classified as a complex item due to the inclusion of the adjectives and the adverb, a difference highlighted within Table 2. The presentation of each sentence was consistent in that all the noun phrases were simple or complex and all verb phrases were either simple or complex; for example, a sentence would not have a simple noun phrase followed by a complex verb phrase. See Table 2 for examples of both complex and simple sentences. During the beginning of training (but not test), the simple and complex stimuli included noun phrases presented without a corresponding verb or adverb. The simple phrases had only a noun and a determiner, while the complex phrases included noun, adjective, and determiner. Figure 2 illustrates all possible word class combinations and identifies the two kinds of phrases and four kinds of sentences that could be generated by the Brocanto2 grammar.

**TABLE 2:** Examples of simple and complex input for klin and praz in Brocanto2.

	**Brocanto2 sentence**	**Word categories**
**Simple input**		
*Klin*^∧^	*Blom lu klin*	*N Det + V*
*Praz*^+^	*Blom lu neep li praz*	*N Det + N Det + V*
**Complex input**		
*Klin*^∧^	*Blom neimo lu klin noyka*	*N Adj Det + V Adv*
*Praz*^+^	*Blom neimo lu neep troise li praz noyka*	*N Adj Det + N Adj Det + V Adv*

**Example sentences from both complexity conditions containing the two verbs that could not be both transitive and intransitive. Noun, N; determiner, Det; verb, V; adjective, Adj; adverb, Adv; ∧denotes intransitive verb and ^+^ denotes transitive verb.**

### Procedure

#### Brocanto2 Artificial Language Learning Paradigm

Participants were taught the Brocanto2 vocabulary to 100% accuracy prior to starting any other aspects of the study. Vocabulary training consisted of a self-paced PowerPoint presentation that paired Brocanto2 audio with the symbols for nouns or a general animation to signify that an action was happening. At no point during the vocabulary training was explicit information given regarding spelling, translations, or parts of speech. The vocabulary assessment was a second PowerPoint presentation that replicated the training PowerPoint with one important difference: participants had to generate the correct Brocanto2 word for each slide. Therefore, participants had to self-generate 100% of the vocabulary before progressing to the next phase of the training.

**FIGURE 3:**
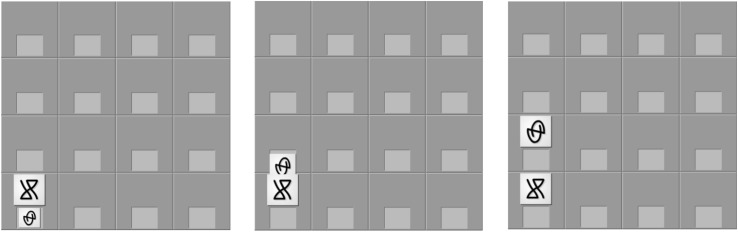
Example of progressive screen shots for an animated movement. The corresponding audio was *neep li vode lu yab* for the simple version of the sentence that represented the move or *neep neime li vode neimo lu yab noyka* for the complex version.

The participants were then presented with game training that consisted of an introduction to the computerized board game they would be playing at a later point, thus providing a meaningful context for the artificial language on which they were subsequently trained^3^. Participants read the rules of the game and viewed the four possible types of game moves (move, switch, capture, or release). They were then asked to practice making each move on the game board by selecting game tokens with a mouse and repeating the move that had just been visually presented, as illustrated by Figure 3. At no point were explicit translations of the symbols or movements provided. After becoming familiar with the rules of the game, participants continued on to language training. Note that all participants received the same vocabulary and game training – it was not part of the manipulation.

Before beginning the task, participants were instructed that they would receive training on an artificial language and would be presented with words, phrases, and sentences that would correspond to still and moving images on the game board. Participants were told they would complete a short quiz to test their memory and they would not be able to review this information again. They were also informed that they would then use the artificial language to play a board game at a later point. No other instructions were given; therefore, training can be viewed as implicit or uninstructed (not incidental) due to the lack of explicit information or explanation of the Brocanto2 language rules. Note, however, that the implicit, uninstructed format of the training does not entail that learning is necessarily implicit in nature.

Participants were pseudorandomly assigned to either simple (*N* = 24) or complex (*N* = 23) input conditions, with every other learner assigned to the simple condition. All participants received training phrases and sentences featuring identical nouns and verbs, presented either in a simple or complex format (100 items). Thirty-six of the training items were phrases, while 64 were sentences.

In the “simple” training condition, 80% of the sentences that participants received were simple while the other 20% were complex; in the “complex” training condition, 80% of the sentences were complex while 20% were simple. This particular ratio of stimuli was utilized so that participants would be exposed to every word category in Brocanto2 and its function in a sentence while still presenting a vast majority of one particular type of stimuli. Furthermore, a 1:4 ratio has also been used in previous cognitive linguistics studies examining the learning and generalization of grammatical regularities for novel verbs (e.g., Casenhiser and Goldberg, 2005).

Participants were presented with the Brocanto2 stimuli aurally and *always* received simple stimuli *before* complex stimuli regardless of the training condition. Each training condition began with the visual presentation of the 36 individual symbols that corresponded to Brocanto2 noun phrases (simple and complex) and progressed to 64 fully animated moves with corresponding sentences (simple and complex). That is, all participants received the training items in the following order: simple phrases, complex phrases, simple sentences, complex sentences. This ordering of phrases being presented before full sentences follows the structure of previous Brocanto2 studies (e.g., Morgan-Short et al., 2012b, 2014) and that of studies exploring the starting small hypothesis (e.g., Kersten and Earles, 2001; Conway et al., 2003; Poletiek et al., 2018). Presentations for each noun phrase consisted of a single, static game piece while the audio was played. An animated movement involving one or more pieces on the game board accompanied the presentation of sentences, and in this case, the audio was played before the animated movement occurred. At the conclusion of each noun phrase or animation, there was a 1 s break before the next item appeared on screen. The game pieces and animations presented to participants were identical across the two conditions—the training only varied in terms of the audio. More specifically, participants in the simple training condition were presented with twenty-nine simple noun phrases followed by seven complex noun phrases, and then fifty-one simple sentences followed by thirteen complex sentences. In the complex condition, the overall order of sentence types would remain the same, but participants would instead be trained on seven simple noun phrases followed by twenty-nine complex noun phrases, and then thirteen simple sentences followed by fifty-one complex sentences.

The primary language assessment in this study consisted of a grammaticality judgment task (GJT). The GJT requires the participant to make a judgment regarding the grammaticality (yes or no) of a sentence and is commonly used across second-language learning literature (cf. Loewen, 2009; Plonsky et al., 2019). The GJT consisted of 72 novel sentences, half (36) of the stimuli were simple sentences and half were complex. Of the 36 simple sentences, half were correct and half contained a violation; this was also the case for the complex sentences. The same set of GJT items was used at each session.

Grammatical sentences for the GJT were novel, i.e., correct sentences that were not presented during training. In general, ungrammatical items were generated by introducing violations in the novel, correct sentences. However, four ungrammatical simple sentences had to be created using violations of sentences that appeared in training due to the limited number of such sentences that could be generated by the grammar. There were an equal number of word order (6), verb argument (6), and gender (6) violations in both the simple and complex GJT stimuli. Word order violations were created by replacing a word from one of the five word categories (e.g., noun) with a word from a different category (e.g., adjective, article, verb, adverb). Verb argument violations were created by replacing a transitive verb with an intransitive verb and vice versa, therefore these violations were constrained to the verbs *klin* and *praz*. Grammatical gender violations were created by replacing a feminine adjective or article with a masculine adjective or article, and vice versa. Violations never occurred on the first or final word, and violation position among words was distributed as evenly as possible. Word frequency within each grammatical category was also as equally distributed as possible across all sentences. Examples of each type of violation sentence can be found in Table 3.

**TABLE 3:** Example correct Brocanto2 sentences and violations thereof.

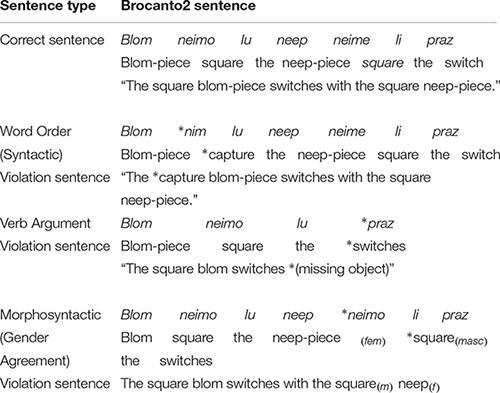

**^∗^Denotes the location of the violation.**

The GJT was programmed in SuperLab 5 and the stimuli (the Brocanto2 sentences) were randomized. The GJT began by guiding participants through the instructions; all directions were presented in white font (size 30) on a black background. The initial screen informed participants that the task was to make a series of judgments regarding new sentences in the artificial language. These judgments were, in order: grammaticality (good or bad), confidence rating (confident or not confident), and source attribution (rule, memory, intuition, or guess). Participants were asked to make each judgment as quickly and accurately as possible. Although the confidence rating and source attribution data is not analyzed for the current study, the full methodology is presented so that the reader fully understands the task demands and to acknowledge that this could have influenced other results (see Brill-Schuetz, 2016, for analyses of the confidence ratings and source attributions).

### Hypotheses and Planned Analyses

This experimental design enables us to examine three separate main hypotheses. The first, that participants will exhibit retention of statistically learned sequences within an artificial language over the course of 2 weeks, will be tested by examining whether or not participants’ accuracy is above chance on the GJT at the second session. In addition, we will examine the degree to which this performance is maintained across sessions, as perfect retention is not expected. As a reminder, participants were trained under what can be considered implicit training conditions, meaning they received repeated exposure to the language without any explicit instruction on the rules of the grammar. The second hypothesis, derived from the “starting-small” literature, is that participants trained on the simpler set of items will outperform their peers in the complex condition overall. Better learning due to the reduced input complexity received in training will lead to better memory both in the short- and long-term for those in the simple training condition. This will be evaluated by looking at the relative performance (accuracy) on the GJT of each group on the GJT at both the first and second sessions. Specifically, participants in the simple training condition should show stronger learning at session one, and this advantage will be carried forward to session two as well.

The third hypothesis consists of multiple parts; the first part of hypothesis three is that the complex training group will rely more on chunk strength when judging the grammaticality of test items than the simple training group; thus, there may be an interaction effect between chunk strength and group. Hypothesis three will be assessed by modeling the relationship between the properties of each test item (e.g., chunk strength) and the likelihood that participants from each group would choose to endorse that item as grammatical. For these analyses we will rely on measures of endorsement rather than accuracy in order to better isolate the aspects of the knowledge participants used to discern between grammatical and ungrammatical test items. Given our interest in better understanding the retention of the grammar embedded within the artificial language, we also wanted to examine how the hypothesized effect of chunk strength on participants’ grammaticality judgments manifested itself across sessions, expecting that its influence might diminish over time. Therefore, the second part of hypothesis three predicts that there will be an interaction between chunk strength and time. We planned to model this interaction using a GLMM, following them up with a series of correlational analyses. Overall, we hope to show that participants in the complex training group rely to a greater extent on chunk strength when endorsing items, and that this reliance changes to some degree over time.

## Results

### Participant Performance and Retention

Before presenting analyses related directly to our hypotheses and research questions, t-tests were conducted to validate that participants exhibited evidence of learning from the two training conditions. As shown in Table 4, those in the simple training condition demonstrated above chance performance at both the first [*t*(23) = 4.018, *p* = 0.001, *d* = 0.83] and second [*t*(23) = 3.835, *p* = 0.001, *d* = 0.75] sessions. Those in the complex condition showed above chance accuracy at session one [*t*(22) = 2.907, *p* = 0.008, *d* = 0.60], but not at session two [*t*(22) = –0.172, *p* = 0.865, *d* = 0.03]. These results are taken to support that learning had taken place in both the simple and complex training condition.

**TABLE 4:** GJT accuracy performance by training condition across sessions.

	**Simple training**		**Complex training**	
		
	**Session 1**	**Session 2**	**Session 1**	**Session 2**
Correct (SD)	59.9% (0.12)	57.5% (0.09)	54.2% (0.07)	49.7% (0.09)
95% CI	54.8–65.0%	53.4–61.5%	51.2–57.1%	45.6–53.7%

**Mean percent correct on the GJT for participants in each training condition at both sessions, along with standard deviations in parentheses. 95% Confidence Intervals are reported beneath each mean. These statistics were calculated by-subject.**

Related to the first hypothesis about retention, overall participant accuracy was above chance (i.e., 50%) when judging items as grammatical or ungrammatical at both sessions one [*t*(46) = 4.774, *p* < 0.001, *d* = 0.69; mean: 56.9% correct; standard deviation: 0.10; 95% CI: 54.1–60.0%] and two [*t*(46) = 2.452, *p* = 0.018, *d* = 0.36; mean: 53.6% correct; standard deviation: 0.10; 95% CI: 50.7–56.6%]. A paired t-test to examine how GJT accuracy degraded between sessions showed that while participants did show above chance performance at session two, it was significantly lower than their performance at session one [*t*(46) = –3.0, *p* = 0.004, *d* = 0.33]. This demonstrates that participants retained knowledge of the pattern of the artificial language’s grammatical regularities over the course of 2 weeks, although this retention was not perfect.

In regard to the second hypothesis about what whether the type of training affected accuracy and retention, we examined how each group of participants performed on the GJT across both sessions. Looking deeper to see what aspects of training affected accuracy and retention, a 2 (session) × 2 (training condition) mixed ANOVA analyzing accuracy showed significant main effects for both session [*F*(1, 45) = 9.058, *p* = 0.004, η*_*p*_*^2^ = 0.168] and training group [*F*(1, 45) = 6.872, *p* = 0.012, η*_*p*_*^2^ = 0.132], while the interaction effect did not reach significance [*F*(1, 45) = 0.796, *p* = 0.377, η*_*p*_*^2^ = 0.017]. In spite of the non-significant interaction term, a follow-up on group differences was performed in order to clarify the different pattern of results found between groups, which should not be over-interpreted. A set of paired t-tests showed that participants in the simple training condition did not exhibit a statistically significant change in performance between sessions [*t*(23) = 1.46, *p* = 0.158, *d* = 0.23], while those in the complex training condition performed significantly better at session one than they did at session two [*t*(22) = 2.84, *p* = 0.009, *d* = 0.55].

While this set of results indicates that those in the complex training condition did not exhibit learning or retention as well as those in the simple training condition, it is also possible that they were sensitive to other aspects of the items besides their grammaticality. That is, it is possible that they learned some features of the training set besides the grammar and used those as cues when accepting or rejecting items. Other analyses that are specific to performance related to test items, complexity (simple vs. complex) and grammatical structure (syntax, morphosyntax, and verb argument), are reported in Brill-Schuetz (2016).

### Modeling Predictors of Item Endorsement

In relation to hypothesis three, in order to get a clearer picture of the type(s) of information to which participants in either group showed sensitivity, we used the dependent variable of endorsement rates rather than accuracy. Endorsement rates were calculated by looking at the proportion of “yes” responses when participants were asked if they thought a GJT test item was grammatical, even when it was not. More specifically, when a participant responded “yes” to either a grammatical or ungrammatical item, they would receive a score of “1” whereas when they responded “no” to either a grammatical or ungrammatical item, they would receive a “0” instead. Endorsement rates for each group at both sessions can be found in Table 5. Using endorsement rates for particular items will allow us to figure out how each group may have used the information they statistically learned when performing the GJT in a way that just looking at the group’s mean performance (percent correct) cannot. For each item, we can connect the chunk strength of that item to the likelihood that it was endorsed by the participants; thus we will be able to determine the sub-features of the items that most strongly led participants to say “yes” and “no” to them when making grammaticality judgments at test.

**TABLE 5:** GJT response patterns by training condition for item endorsement across sessions.

	**Simple training**		**Complex training**	
		
	**Session 1**	**Session 2**	**Session 1**	**Session 2**
Grammatical (SD)	64.4% (0.13)	64.4% (0.14)	55.6% (0.28)	58.0% (0.17)
95% CI	60.3–69.0%	59.6–69.1%	46.1–65.1%	52.2–63.9%
Ungrammatical (SD)	45.5% (0.15)	49.5% (0.17)	48.2% (0.20)	58.5% (0.17)
95% CI	40.4–50.5%	43.8–55.2%	41.6–54.9%	52.7–64.4%

**Endorsement rates on the GJT for participants in each training condition at both sessions for both grammatical and ungrammatical items, along with standard deviations in parentheses. 95% Confidence Intervals are reported beneath each mean. These statistics were calculated by-item.**

After calculating these endorsement rates, we investigated what fixed factors were the strongest predictors of item endorsement. To do this we used a series of generalized linear mixed effect models (GLMMs) to examine the effects of training condition, chunk strength, and time (session) on item endorsement using the LME4 package in R (Bates et al., 2014). The model included as fixed effects: training group (complex vs. simple), chunk strength of GJT item (continuous), and time (session one vs. session two). We included as a random effect the intercepts for GJT endorsement by subject. This controlled for individual differences in response bias, making it easier to detect fixed effects of our variables of interest.

The chunk strength of each item was calculated in order to determine the extent to which the participants used this kind of fragment information when endorsing items. The chunk strength referenced here was measured as the sum of the frequency of occurrence in the training items of each of the fragments in a test item, weighted by the number of fragments in that item (Knowlton and Squire, 1994). For example, the associative chunk strength of the item ZVX would be calculated as the sum of the frequencies of the fragments ZV, VX, and ZVX divided by 3. A higher number indicates that a test item is well supported by chunk information in the training items. Chunk strength thus captures the repeated use of 2- and 3-element chunks in a sequence, allowing for generalization from known sequences to novel ones. So, just because a test item did not occur in training that does not mean that some portion of it did not appear as part of a training item. If “the brown cat” is a training item while “the brown cow” is a test item, the chunk “the brown” appeared in both, and therefore would contribute to the chunk strength of the test item.

With the sets of training and test items used in this study, chunk strength actually was significantly greater for grammatical vs. ungrammatical over all test items, meaning that it was a potentially useful cue for performing accurately on this task for both the simple [*t*(70) = 2.268, *p* = 0.026, *d* = 0.53] and complex [*t*(70) = 2.396, *p* = 0.019, *d* = 0.56] training groups; this was coincidental, as chunk strength was not factored in when creating the stimuli for this experiment. Note that these comparisons were computed separately given that the two groups had different training sets, even though the test sets were exactly the same. Descriptive statistics for the chunk strength of both grammatical and ungrammatical test items for each training group can be found in Table 6.

**TABLE 6:** Average chunk strength of test items for each training group.

	**Simple training**		**Complex training**	
		
	**Grammatical**	**Ungrammatical**	**Grammatical**	**Ungrammatical**
CS (SD)	6.93 (3.74)	5.02 (3.40)	7.34 (3.79)	5.36 (3.20)
95% CI	5.67–8.20	3.87–6.17	6.05–8.62	4.27–6.44

**Mean chunk strength of grammatical and ungrammatical test items for each training condition, along with standard deviations in parentheses. 95% Confidence Intervals are reported beneath each mean. CS, chunk strength.**

The initial model (Model 1) with separate fixed effects is reported in Table 7. However, due to the nature of the manipulation and the variables of interest, another model with three two-way interaction terms was built. This model (Model 2) was primarily built in order to appropriately control for the two-way interactions’ inclusion in the final model containing the key three-way interaction term. Additionally, we hypothesized that the effect of chunk strength on item endorsement may potentially degrade with time due to the nature of memory, thus we included an interaction term between these variables. The results for Model 2, which include these interaction terms, are also reported in Table 7. To test if the inclusion of interaction terms improved upon Model 1, a deviance test was conducted (Singer and Willett, 2003). The interaction terms improved model fit, χ^2^(3) = 76.681, *p* < 0.0001.

**TABLE 7:** Summaries of the two generalized linear mixed effects models.

**Fixed Effects**	**Model 1**	**Model 2**	**Model 3**
Intercept	–0.847^∗∗∗^	–1.961^∗∗∗^	–2.527^∗∗∗^
	(0.134)	(0.217)	(0.268)
Group	0.095	1.093^∗∗∗^	2.100^∗∗∗^
	(0.137)	(0.229)	(0.357)
Chunk	0.126^∗∗∗^	0.281^∗∗∗^	0.374^∗∗∗^
	(0.007)	(0.025)	(0.036)
Time	0.180^∗∗∗^	0.682^∗∗∗^	1.05^∗∗∗^
	(0.052)	(0.118)	(0.155)
Group^∗^Chunk		–0.110^∗∗∗^	–0.279^∗∗∗^
		(0.015)	(0.048)
Group^∗^Time		−0.227^∗^	–0.887^∗∗∗^
		(0.105)	(0.207)
Chunk^∗^Time		–0.064^∗∗∗^	–0.125^∗∗∗^
		(0.015)	(0.022)
Group^∗^Chunk^∗^Time			0.111^∗∗∗^
			(0.030)
**Random Effects**			
Subject (Intercept)	0.189	0.192	0.193
	(0.435)	(0.438)	(0.439)
**Goodness Of Fit**			
Log likelihood	−4231.2	−4192.9	−4186.0
AIC	8472.5	8401.8	8390.1
BIC	8506.4	8456.0	8451.1

**Estimated coefficients are listed while standard errors are reported in parentheses. ^∗^p < 0.05, ^∗∗∗^p < 0.001.**

**FIGURE 4:**
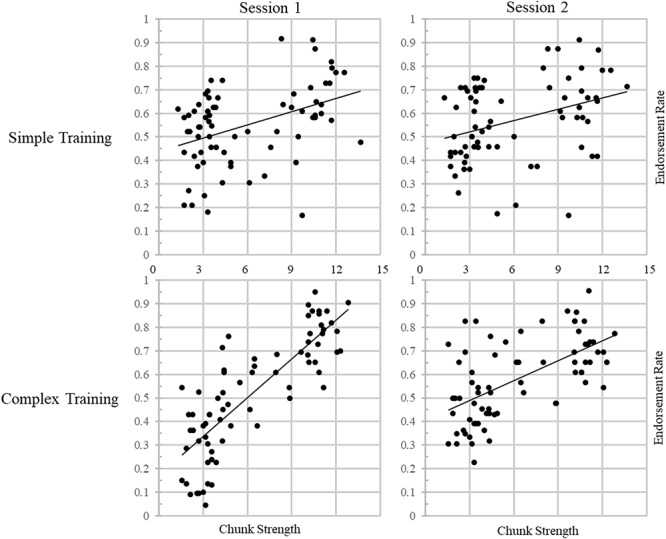
Endorsement rates correlated with chunk strength across sessions for each training group, illustrating the three-way interaction effect in Model 3. Trend lines represents linear lines of best fit.

A further desire to also include a potential three-way interaction between training condition, session, and chunk strength led to the creation of Model 3. This model outperformed Model 2 [χ^2^(1) = 13.716, *p* = 0.0002], supporting the hypothesis that the effect of training on retention would differ between groups. Importantly, we know that the three-way interaction term is solely responsible for the improvement in the model’s fit due to the inclusion of all three two-way interaction terms in Model 2. Visual inspection of Figure 4 demonstrates this interaction nicely, showing that the effect of chunk strength on item endorsement decreases over time and illustrating the greater impact of chunk strength on endorsement for participants in the complex training condition; additional correlational analyses will attempt to verify the directionality of the interaction. Note that including Item as a random effect resulted in a model that failed to converge when also including the critical three-way interaction.

With the aim of extending the GLMM’s findings, we also chose to examine the ways in which accuracy and endorsement varied depending on the surface-level features of each test item at both sessions within either training group. In order to do so, we conducted subsequent analyses on by-items data rather than collapsing across participants. As described in the methods, this meant that the twenty-three participants in the complex training condition and 24 in the simple training condition constituted the number of observations across the seventy-two test items, and due to the differing fragment statistics for each training condition, all subsequent analyses treated these groups separately.

To further explore the results of the GLMMs, traditional, frequentist analyses were conducted. Both training groups exhibited a correlation between an item’s chunk strength and their endorsement rate. Notably, while the simple training group showed small to moderate correlations at both sessions one (*r* = 0.409, *p* < 0.001) and two (*r* = 0.342, *p* = 0.003), the complex training group showed an extremely strong correlation at session one (*r* = 0.819, *p* < 0.001), as well as a moderately strong correlation at session two (*r* = 0.598, *p* < 0.001), suggesting that this pattern drove the three-way interaction above. A Fisher’s r to z comparison of these correlation coefficients shows that the two groups’ correlations are significantly different from one another at both sessions one (*z* = –4.23, *p* < 0.001) and two (*z* = –1.96, *p* = 0.05).

To verify the validity of these contrasts, we examined whether there was inherently a stronger relationship between the test items’ chunk strength and their grammaticality for the complex group than for the simple group. If that were the case, then the meaningfulness of the difference between the groups’ correlations would be reduced – it would have just been the case that for one group these two variables tracked one another more closely and was not driven by the differential effects of their training. However, this was not the case, as the mean chunk strength of grammatical items was not significantly different between the simple and complex training conditions [*t*(70) = –0.456, *p* = 0.649, *d* = 0.11], a pattern that also held true for ungrammatical items [*t*(70 = –0.429, *p* = 0.670, *d* = 0.10]. Refer back to Table 6 to find the relevant means, standard deviations, and confidence intervals. This shows that chunk strength was not a stronger cue for either group of participants, suggesting that the complex group’s reliance on it was not merely because it was more useful for them in terms of differentiating grammatical and ungrammatical items at test.

A key difference between training groups also emerged when looking at how the chunk strength of each item correlated with participants’ accuracy when judging the grammaticality of that item. Only participants in the complex training condition showed a statistically significant relationship between accuracy and chunk strength, and they did so at session one (*r* = 0.300, *p* = 0.010), as well as at session two (*r* = 0.248, *p* = 0.035), whereas those in the simple training condition did not at either session one (*r* = 0.187, *p* = 0.116) or session two (*r* = 0.139, *p* = 0.244). This underscores the complex training group’s reliance on the surface level properties of the test stimuli when engaged in the GJT.

## Discussion

The set of results described above demonstrates that first, learners overall seem to be able to retain the regularities of an artificial language over the span of 2 weeks. While retention was not perfect, as performance degraded over time, a sufficient degree of knowledge was maintained to show a learning effect at the second test session. This is a longer time interval than what is typically found in the extant literature on SL, which typically only looks at retention after a period of hours or days. Extensive research on other types of learning and memory has found that participants can recall learned items at rather long intervals (Tulving et al., 1982; Schacter, 1987; Roediger, 1990; Mitchell, 2006). Note that the test items in this study were not present during training and were only seen once previously during a test session using a randomized presentation, where half of the trials were foils. This suggests that instead of recalling previous answers, participants were able to use learned knowledge to respond to test items.

The ability of participants to retain their knowledge of statistically learned dependencies over time is crucial to understanding the way in which experience with linguistic constructions affects later processing (Reali and Christiansen, 2007; Wells et al., 2009). In order for SL to impact language processing in the way it has long been hypothesized (Saffran, 2001), the learned statistical patterns must be retained in memory. Our findings demonstrate that such retention is possible and adds support for such theories. Determining the limits of retention for statistically learned regularities should be a priority for future research, as the SL literature has long rested on the assumption that such associations form a key foundation for language learning.

There is an interesting pattern of results that speak to both the SL literature (directly above) and the “starting small” theories. Firstly, the fact that those trained extensively on simple items exhibited above-chance accuracy performance at both sessions provides evidence that “starting small” with extensively scaffolded, staged training leads to more accurate learning and retention of grammatical regularities – their performance did not show a statistically significant decline between sessions. Whereas both training conditions within the present study started small, participants in the simple training condition were given significantly more time to learn from the simpler items. Intentionally reducing the problem space for learners during the early phases of acquisition seemed to improve learning outcomes in this study (see also Conway et al., 2003). Poletiek et al. (2018) have recently demonstrated that participants are able to use their memory of previously encoded, simple structures to facilitate their learning of newer, more complex ones. They also point out the importance of incrementally exposing learners to increasingly complex items, rather than simply longer ones.

The present research also shows a similar trend to other studies that demonstrate how overrepresenting simplified input early on during training can lead to improved learning (Pine, 1994; Perfors et al., 2011). Scaffolding reflects the way in which young learners typically acquire language, however, the results here suggest that forcing adults to adopt more immature strategies when learning a novel language may confer benefits. Future research into the relationship between second language learning in adults and intentionally constrained input could be important to shaping adult pedagogical strategies and our understanding of language acquisition more generally.

Conversely, participants in the complex training condition showed above-chance accuracy on grammaticality judgments in session one, yet they did not match the performance of the simple training group. However, what is interesting is that participants in the complex training condition showed evidence of relying more heavily on chunk strength, which captures basic frequency information. While this suggests that the complex training condition promoted simple learning of frequency patterns, it may not have been enough exposure for participants to induce the more complex probabilistic patterns underlying grammatical regularities. Future research may increase the length of exposure to complex stimuli to investigate if this does improve overall performance.

The overall set of findings fits in well with recent proposals about how the constraints placed on learning by our cognitive abilities shape the way in which we process, and thereby learn, language (Christiansen and Chater, 2008, 2016). The proposed “Now-or-Never bottleneck” refers to the process by which language users must continuously recode and compress linguistic input in order to keep up with comprehension. In this framework, language processing *is* language learning; during comprehension, we must effectively process the input as quickly and accurately as possible before it is overwritten or interfered with by new incoming information. Learners take the information that makes it through the bottleneck as far as they can – in the simple training condition of the present study, more exposure to simple items may have allowed them to process subregularities more efficiently and thereby better deal with similar patterns in the more complex items, whereas those in the complex condition were only able to rely on the more surface-level information contained within the chunks that they learned and retained.

In sum, participants in this study showed the ability to retain information learned within an artificial language learning paradigm over the course of 2 weeks. It also appears that increasing exposure to simplified grammatical structures in beginning stages of learning confers benefits to adult learners. Importantly, some of the grammatical regularities of this artificial language are retained in long-term memory in a way that has not been shown previously in SL research. This falls in line with theories about both first- and second-language acquisition, and also with new ideas concerning the role of processing constraints on language learning. Overly challenging and complex input seems to derail learners and affects the kind of information they are sensitive to, leading them to rely more on simple fragment frequency rather than higher-order associations between them. This pattern of results contrasts with learners who were provided scaffolded input, as they demonstrated better acquisition of the higher-order regularities and relied less on basic frequency cues when choosing to endorse items as grammatical or ungrammatical.

## Data Availability Statement

The datasets generated for this study are available on request to the corresponding author.

## Ethics Statement

The studies involving human participants were reviewed and approved by UIC Institutional Review Board (protocol 2008–0496). The patients/participants provided their written informed consent to participate in this study.

## Author Contributions

KB-S and KM-S conceived of the original study. KB-S collected the data. EJ, KB-S, KM-S, and MC conceived of the current analysis, analyzed the data, and revised the manuscript. EJ wrote the initial draft.

## Conflict of Interest

The authors declare that the research was conducted in the absence of any commercial or financial relationships that could be construed as a potential conflict of interest.

## Footnotes

1 This research was approved by the University Human Subjects Institutional Review Board under protocol 2008–0496.

2 Brocanto2 is available upon request by email to karams@uic.edu.

3 Participants played the computerized board game as part of a comprehension assessment that followed the GJT, the results of which are not reported here.
